# Uptake and factors associated with HIV self-testing among women in South Africa

**DOI:** 10.4102/jcmsa.v2i1.21

**Published:** 2024-05-10

**Authors:** Michael Ekholuenetale, Osaretin C. Okonji, Chimezie I. Nzoputam, Amadou Barrow

**Affiliations:** 1 Department of Epidemiology and Medical Statistics, Faculty of Public Health, College of Medicine, University of Ibadan, Ibadan, Nigeria; 2 School of Pharmacy, University of the Western Cape, Cape Town, South Africa; 3 Department of Medical Biochemistry, School of Basic Medical Sciences, University of Benin, Benin City, Nigeria; 4 Department of Public and Environmental Health, School of Medicine and Allied Health Sciences, University of The Gambia, Brikama, Gambia

**Keywords:** HIV and/or AIDS, sub-Saharan Africa, women, sexually transmitted infection, South Africa

## Abstract

**Background:**

Human immunodeficiency virus (HIV) self-testing (ST) is a convenient and discreet practice to know HIV status. It is required to reach the underserved population subgroups. We examined the uptake and factors associated with HIVST among reproductive-aged South African women.

**Methods:**

A sample of 8182 women from 2016 South African Demographic and Health Survey data were analysed. Percentage and multivariable logistic regression model were conducted. The significance level was set at *p* < 0.05.

**Results:**

Approximately 3.1% of women had HIVST uptake. Respondents with primary education had a 75% reduction in the odds of HIVST, compared with women who had no formal education. Women who are employed were 1.44 times as likely to have HIVST uptake compared to those not employed. Women aged 20–24 years, 25–29 years, 30–34 years, and 40–44 years had about two times higher odds of HIVST, when compared with women aged 15–19 years. Women who read newspaper or magazine less than once a week or at least once a week were 1.90 and 2.25 times as likely to have HIVST uptake, respectively, when compared with those who do not read at all.

**Conclusion:**

The prevalence of HIVST uptake was low. HIVST was associated with women’s sociodemographic characteristics. The findings highlight the importance of addressing women’s needs to know their HIV status.

**Contribution:**

This study adds to the body of literature in understanding the utilisation patterns of HIVST across women in South Africa.

## Introduction

In sub-Saharan Africa (SSA), adolescent girls and young women are at an increased risk of human immunodeficiency virus (HIV) infection than their male counterparts. In 2020, women and adolescent girls accounted for nearly two-thirds (63%) of all new HIV infections in sub-Saharan Africa.^1^ Human immunodeficiency virus was a top cause of death for women of reproductive age (15–49 years) in 2017.^2^ In SSA, more than 4000 adolescent girls and young women (age 15–24 years) were infected with HIV every 7 days in 2020.^3^ This age group of women also accounts for 26% of new HIV infections in the Eastern and Southern African region.^4^ South Africa remains excessively burdened by the HIV pandemic, as there is a rapid increase in the number of people living with HIV in the country, from an estimated 3.8 million people in 2002 to as high as 8.2 million people in 2021.^5^ The HIV epidemic in South Africa, just as in many other Southern African countries, remains concentrated among the higher-risk subgroups, particularly among women in their reproductive years. There is an estimate of 26.3% HIV prevalence among reproductive-aged women (15–49 years) in South Africa compared to HIV prevalence of 14.8% in men.^6^ Numerous factors influence the susceptibility of women to HIV; these include socioeconomic, biological, cultural, structural and behavioural risks.^7^

Despite the high burden and high prevalence and incidence of HIV contamination among women in their reproductive age, uptake of HIV testing in this population is suboptimal.^6,8^ A recent survey conducted in South Africa revealed that 36% of women between 15 and 24 years were ignorant of their HIV status.^6^ By 2030, the Joint United Nations Programme on HIV and AIDS (UNAIDS) has established a global 95-95-95 objective of reaching 95% of adults who are aware of their HIV status, 95% of HIV-positive individuals who are engaged in antiretroviral (ART), and 95% of those on ART who have viral suppression.^9^ Although South Africa made an effort towards achieving the UNAIDS 90-90-90 targets, many HIV-positive people still did not know their HIV status.^6^ Several techniques have been used to deliver HIV testing services (HTS) to women of reproductive age, including embedding HTS within family planning or prenatal care programmes. However, many young women only get to know their HIV status during their first antenatal services, resulting in delay in testing, diagnosis and uptake of treatment.^10^

The World Health Organization has advocated HIV self-testing (HIVST) as a significant alternative to existing HIV counselling and testing (HCT) procedures, and many countries, including South Africa, have already included HIVST as a component of testing strategy.^11^ HIV self-testing knowledge and use remain low in South Africa as less than a quarter of the populace have heard of it and only 3% have used it.^12^ HIV self-testing acceptability rate ranges between 22% and 94%, with men having a higher HIVST acceptability rate than women in sub-Saharan Africa.^13^ Individuals living in urban areas, those highly educated and wealthy, have greater awareness of HIVST.^12^ It has been shown to have the potential to increase HIV testing uptake and frequency^14,15^ because it has the benefit of privacy and could help to reduce structural barriers that influence HIV testing. Another study reported that HIVST acceptability was higher among women.^16^ While men preferred HIVST because of convenience and efficiency, women favoured HIVST as a result of its potential to provide autonomy and empowerment.

Several factors influence HIVST uptake among women; these include age, marital and parental status, educational status, wealth status, prior contact with HIV testing, and HIV uptake at the household levels.^17^ Determining the factors influencing the uptake of HIVST among women in their reproductive age is crucial, considering the added worth of HIVST programmes in closing the gaps of HIV testing coverage. Very few population-based researchers have inspected factors influencing HIVST uptake. This study, therefore, assessed the factors associated with HIVST uptake among reproductive-aged South African women and provided recommendations that would assist in facilitating HIVST use among key populations.

## Methods

### Data source

The authors utilised cross-sectional data from the South African Demographic and Health Survey (DHS) of 2016 to create a nationally representative sample. Data from 8182 women of reproductive age were used for this study. A previous article has reported the data extraction approach.^18^ The data are available on the public space and can be accessed at: http://dhsprogram.com/data/available-datasets.cfm. Details of the DHS sampling procedure have been previously reported.^19^

### Variables selection and measurement

#### Outcome

The dependent variable was based on the question on use of HIV test kits; women who responded ‘has tested with HIV test kits’ were coded ‘1’, while those who responded ‘never used HIV test kits’ were coded ‘0’.

#### Independent variables

A previous article has reported the independent variables used in this study.^18^

### Statistical analysis

A previous article has reported statistical analysis approach.^18^ Stata version 14 (StataCorp., College Station, Texas, United States) was utilised for data analysis.

### Ethical considerations

Ethics approval for this study was not required because the data are secondary and is available in the public domain. More details regarding DHS data and ethical standards are available at: http://dhsprogram.com/data/available-datasets.cfm.

## Results

**FIGURE 1:**
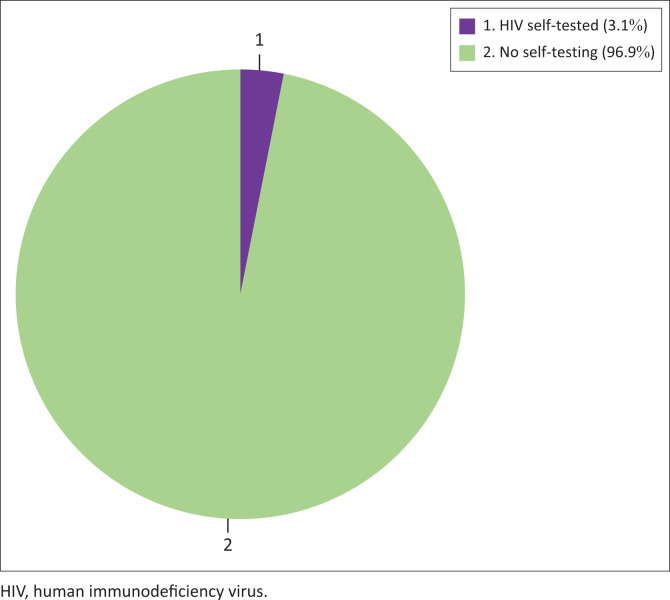
Uptake of HIV self-testing among South African women.

Approximately 3.1% (95% CI: 2.6% – 3.7%) of South African women had HIVST uptake as shown in Figure 1.

Table 1 shows the prevalence of HIVST across the levels of women’s characteristics. Approximately 8.5% of women with higher education had HIVST uptake. About 5.5% of those from richest household had HIVST uptake. Few urban women (3.5%), employed (4.6%), read newspapers or magazines (4.5%), listened to radio (3.7%) or watched television at least once a week (3.5%) had HIVST uptake, respectively. The results are summarised in Table 1.

**TABLE 1:** Distribution of HIV self-testing among South African women (*N* = 8182).

Variable	*n*	%	Prevalence of HIVST	95% CI	*p*
**HIV general knowledge**	-	-	-	-	0.710
Poor	2568	31.4	3.3	2.3–4.7	-
Good	5614	68.6	3.0	2.5–3.7	-
**Education**	-	-	-	-	< 0.001*
No formal education	176	2.2	4.9	1.5–14.4	-
Primary	816	10.0	0.9	0.4–1.8	-
Secondary	6335	77.4	2.5	2.0–3.0	-
Higher	855	10.4	8.5	6.6–10.9	-
**Household wealth**	-	-	-	-	0.001*
Poorest	1530	18.7	2.1	1.3–3.6	-
Poorer	1743	21.3	2.4	1.5–3.8	-
Middle	1818	22.2	3.1	2.2–4.3	-
Richer	1665	20.4	2.7	1.9–3.7	-
Richest	1426	17.4	5.5	4.1–7.3	-
**Residential status**	-	-	-	-	0.010*
Urban	4653	56.9	3.5	2.8–4.3	-
Rural	3529	43.1	2.3	1.8–2.9	-
**Employment**	-	-	-	-	< 0.001*
No	5500	67.2	2.3	1.8–2.9	-
Yes	2682	32.8	4.6	3.6–5.8	-
**Age (in years)**	-	-	-	-	0.010*
15–19	1413	17.3	1.1	0.6–1.9	-
20–24	1356	16.6	3.7	2.5–5.2	-
25–29	1352	16.5	3.5	2.5–5.2	-
30–34	1247	15.2	4.3	3.1–5.9	-
35–39	1001	12.2	3.1	2.0–5.0	-
40–44	929	11.4	3.0	1.9–4.7	-
45–49	884	10.8	2.8	1.6–4.7	-
**Region**	-	-	-	-	0.212
Eastern Cape	1024	12.5	1.9	1.1–3.0	-
Free State	839	10.3	4.6	3.2–6.6	-
Gauteng	807	9.9	3.6	2.4–5.4	-
KwaZulu-Natal	1273	15.6	2.8	1.8–4.3	-
Limpopo	1025	12.5	3.3	2.3–4.7	-
Mpumalanga	1048	12.8	2.8	1.9–4.2	-
Northern Cape	688	8.4	2.2	1.4–3.3	-
North West	852	10.4	4.1	3.1–5.5	-
Western Cape	626	7.7	2.6	1.5–4.5	-
**Ethnicity**	-	-	-	-	0.879
African or black people	7070	86.4	3.2	2.6–3.8	-
White people	200	2.4	3.5	1.2–9.4	-
Mixed race people	821	10.0	2.4	1.5–4.0	-
Indian or Asian people	88	1.1	2.7	1.1–6.5	-
Other	3	0.1	-	-	-
**Frequency of reading newspaper or magazine**	-	-	-	-	< 0.001*
Not at all	3074	37.6	1.5	1.0–2.2	-
Less than once a week	2161	26.4	3.1	2.3–4.2	-
At least once a week	2947	36.0	4.5	3.6–5.5	-
**Frequency of watching television**	-	-	-	-	0.001*
Not at all	1444	17.7	1.4	0.8–2.2	-
Less than once a week	778	9.5	3.1	1.9–4.9	-
At least once a week	5960	72.8	3.5	2.9–4.2	-
**Frequency of listening to radio**	-	-	-	-	0.011*
Not at all	2517	30.8	2.2	1.6–3.0	-
Less than once a week	1330	16.3	2.8	2.0–4.0	-
At least once a week	4335	53.0	3.7	3.0–4.5	-
**Marital status**	-	-	-	-	0.146
Never in union	4905	60.0	2.7	2.1–3.4	-
Currently in union/living with a man	2752	33.6	3.8	2.9–4.9	-
Formerly in union	525	6.4	3.4	1.8–6.3	-
**Family motility (years)**	-	-	-	-	0.002*
< 5	1720	21.0	4.6	3.4–6.1	-
Native (5+)	6462	79.0	2.7	2.2–3.3	-
**Gender of household head**	-	-	-	-	0.123
Male	3470	42.4	3.5	2.8–4.4	-
Female	4712	57.6	2.8	2.2–3.5	-

CI, confidence interval; HIVST, human immunodeficiency virus self-testing.

* , Significant at *p* < 0.05.

Table 2 shows the factors associated with HIVST among South African women. Women with primary education had 75% reduction in HIVST, when compared with women with no formal education (OR = 0.25; 95% CI: 0.08–0.72). Women who are employed were 1.44 times as likely to utilise HIVST, when compared with those not employed (OR = 1.44; 95% CI: 1.09–1.92). Older women had higher odds of HIVST. Women who read newspaper or magazine less than once a week or at least once a week were 1.90 (OR = 1.90; 95% CI: 1.28–2.84) and 2.25 (OR = 2.25; 95% CI: 1.53–3.31) times as likely to utilise HIVST, respectively, when compared with those who do not read at all. See details in Table 2.

**TABLE 2:** Factors associated with HIV self-testing among South African women.

Variable	Adjusted odds ratio	95% CI	*p*
**Education**		
No formal education	1.00	-	-
Primary	0.25	0.08–0.72	0.011*
Secondary	0.44	0.18–1.05	0.063
Higher	1.01	0.41–2.51	0.976
**Household wealth**		
Poorest	1.00	-	-
Poorer	0.86	0.52–1.41	0.542
Middle	1.12	0.69–1.80	0.650
Richer	0.91	0.55–1.50	0.703
Richest	1.40	0.85–2.29	0.186
**Residential status**		
Urban	1.00	-	-
Rural	0.89	0.67–1.18	0.417
**Employment**		
No	1.00	-	-
Yes	1.44	1.09–1.92	0.011*
**Age (in years)**		
15–19	1.00	-	-
20–24	2.29	1.30–4.04	0.004*
25–29	2.05	1.15–3.66	0.015*
30–34	2.56	1.44–4.56	0.001*
35–39	1.79	0.96–3.33	0.068
40–44	1.88	1.00–3.54	0.049*
45–49	1.42	0.72–2.81	0.311
**Frequency of reading newspaper or magazine**		
Not at all	1.00	-	-
Less than once a week	1.90	1.28–2.84	0.002*
At least once a week	2.25	1.53–3.31	< 0.001*
**Frequency of watching television**		
Not at all	1.00	-	-
Less than once a week	1.44	0.78–2.66	0.246
At least once a week	1.32	0.80–2.19	0.273
**Frequency of listening to radio**		
Not at all	1.00	-	-
Less than once a week	1.01	0.66–1.56	0.959
At least once a week	0.90	0.64–1.27	0.554
**Motility (years)**		
< 5	1.00	-	-
Native (5+)	0.76	0.57–1.02	0.070

CI, confidence interval.

* , Significant at *p* < 0.05.

Table 3 shows the predictive marginal effect of HIVST by women’s characteristics. To determine the influence of women’s factors on HIVST, a marginal predictive analysis was performed. Based on the predictive marginal effects results, we would expect 5.8% of HIVST if the distribution of all characteristics stayed same but every woman had a higher degree. However, if every woman came from the richest household wealth quintile, or dwell in urban residence, we would expect 4.0% or 3.2% of HIVST, respectively. Moreover, if every woman was employed, read newspaper or magazine, listen to radio, watch television at least once a week, we would expect 3.7%, 3.9%, 3.0% and 3.1% of HIVST, respectively. The details of the predictive marginal interaction effects of HIVST are shown in Table 3.

**TABLE 3:** Marginal effect of HIV self-testing among South African women.

Variable	Marginal effect	95% CI	*p*
**Education**			
No formal education	5.7	1.2–10.2	< 0.001*
Primary	1.5	0.4–2.5	< 0.001*
Secondary	2.6	2.2–3.0	< 0.001*
Higher	5.8	4.3–7.2	< 0.001*
**Household wealth**			
Poorest	2.9	1.8–4.0	< 0.001*
Poorer	2.5	1.7–3.3	< 0.001*
Middle	3.2	2.4–4.0	< 0.001*
Richer	2.6	1.9–3.4	< 0.001*
Richest	4.0	3.0–4.9	< 0.001*
**Residential status**			
Urban	3.2	2.7–3.7	< 0.001*
Rural	2.9	2.3–3.5	< 0.001*
**Employment**			
No	2.6	2.2–3.1	< 0.001*
Yes	3.7	3.0–4.4	< 0.001*
**Age (in years)**			
15–19	1.7	0.8–2.5	< 0.001*
20–24	3.7	2.6–4.7	< 0.001*
25–29	3.3	2.4–4.2	< 0.001*
30–34	4.1	3.0–5.1	< 0.001*
35–39	2.9	1.9–3.9	< 0.001*
40–44	3.0	2.0–4.1	< 0.001*
45–49	2.3	1.3–3.3	< 0.001*
**Frequency of reading newspaper or magazine**			
Not at all	1.8	1.3–2.3	< 0.001*
Less than once a week	3.3	2.6–4.1	< 0.001*
At least once a week	3.9	3.2–4.6	< 0.001*
**Frequency of watching television**			
Not at all	2.4	1.3–3.5	< 0.001*
Less than once a week	3.4	2.1–4.7	< 0.001*
At least once a week	3.1	2.7–3.6	< 0.001*
**Frequency of listening to radio**			
Not at all	3.3	2.4–4.1	< 0.001*
Less than once a week	3.3	2.3–4.3	< 0.001*
At least once a week	3.0	2.5–3.4	< 0.001*
**Motility (years)**			
< 5	3.7	2.9–4.6	< 0.001*
Native (5+)	2.9	2.5–3.3	< 0.001*

CI, confidence interval.

* , Significant at *p* < 0.05.

## Discussion

We examined HIV self-testing among South African women using nationally representative large dataset. Our results show approximately 3.1% of HIVST among South African women. This is lower than the overall prevalence of HIV testing reported by a study in east Africa, which was 66.9%,^20^ albeit this varied substantially by location. The documented geographical disparities in HIV testing in eastern Africa^21,22^ could be because of regional differences in quality and availability of HIV testing facilities, as well as HIV and/or AIDS awareness. This study’s prevalence was consistent with a report from Kenya.^23^ This could be because of the fact that both studies utilised community-based study designs, involving youthful and adolescent age groups. This study’s finding on the prevalence of HIVST uptake among South African women shows that more efforts are required to promote the practice. In a previous study, healthcare users had a reasonable knowledge of HIVST and there were indications that they would utilise it if it could be properly managed and made widely available to the public.^24^ On the other hand, the low prevalence of HIVST coverage among the study population may be because of a lack of health policy promoting HIVST that can be implemented among the key population.^25^

There are several barriers to HIVST; these include the lack of policies and health programmes that can enhance access and use of HIVST especially among the key population.^25^ As a result of the low prevalence of HIVST observed in this study, it is recommended that existing healthcare and communal-based systems of HIVST be complemented with policies and programmes that will promote service use.^26^ Institution of such HIVST programmes could have positive effects on the populations’ approach to HIVST uptake. It can also enhance decision making and the ability to choose better approach for HIV testing, timely diagnosis, taking of management actions and precaution particularly with regard to those in hard-to-reach communities and groups.

Previous studies have reported that higher education and literacy have a positive impact for higher HIVST uptake.^17,27,28^ Literacy, enlightenment and higher-level education have been variously reported to influence positively, healthcare decision making and uptake of healthcare services especially among women of reproductive age. This study also found out that women with employment and those from the richest background or household had an increased rate or higher prevalence of HIVST than those without employment and from a poor household. Employed women were found to be about 1.5 times more likely to go for HIVST when compared with those who were not employed. This is in agreement with other studies indicating that the cost of testing^28,29^ and household wealth^30^ significantly influence women’s choice and uptake of HIVST, along with other demographic group such as men counterparts. Educated women are known to have higher odds of financial independence and can make informed health decisions without reliance on their husbands or partners. This study reinforced the significance of education as a means of empowerment, which facilitates women’s autonomy in making decisions regarding HIV self-testing.

The role of higher education and literacy, outcomes of enlightenment or exposure to educational materials, is crucial in the context of healthcare service uptake and utilisation.^31^ Education and enlightenment foster boldness, self-expression and independence.^31,32^ Our study found out that women who read newspaper at least once in a week or less than once in a week are 2.3 and 1.9 times, respectively, more likely to obtain HIVST than those who do not read newspaper or magazine at all. Higher levels of education have been part of the factors that give an individual the ability to seek for better health.^33^ It also gives an individual opportunity for financial freedom as those with higher levels of education are more likely to have employment than those without education.^34,35^ Thus, educated individuals are in a better position to access healthcare services without the immediate concern for financial implications, unlike the less educated counterparts who might lack the financial means. Therefore, it is imperative that efforts are made to provide these healthcare services free of charge or without cost especially among those with poor financial background, unemployed individuals and people in the rural areas who are more likely to be less educated, poor and financially constrained.

Our result was able to show that age of respondents was an HIVST associated factor. It was observed that women who were between the ages of 20 and 44 years had a higher prevalence of HIVST when compared with those aged below 20 years and those who are 45 years and above. Older age has been reported to be a barrier to HIVST compared with those who were young.^36,37,38^ Individuals within this demographic are often described as go-getters and may be more exposed to new information and experiences compared with adolescents and those over 50 years of age. This exposure advantageously positions them to access and acquire knowledge about health-related issues, including HIVST, more readily than other age groups. Given this, it is crucial to focus more attention on adolescents and older adults in terms of awareness creation and mobilisation of HIVST, along with other health-related concerns. It is well acknowledged that older individuals, particularly those above 60 years and adolescents face greater vulnerabilities and disadvantages in accessing healthcare services. Therefore, targeted efforts to engage these groups are essential to ensure equitable access to HIVST and to address the health needs of the entire population effectively.

Our marginal predictive analysis conducted to decode the effects that women’s factors have on HIVST, shows that if all the distribution of the women’s factors are assumed to be the same, but every woman is educated to the level of tertiary levels, we would anticipate that about 6% of the women would have HIVST. Similarly, if we assume that all the study participants were to come from the richest households, reside in urban areas, are employed and engaged in regular enlightenment activities such as reading newspapers or magazines at least once a week, listening to the radio and watching television at least once weekly, it suggests a potential increase in HIVST uptake by 3% – 4%. This shows that higher education, richest household, urban residence and enlightenment positively influence the uptake of HIVST among South African women but less so than if all women had tertiary education.

### Strength and limitations

This study used nationally representative data, which is suitable for making plausible comparisons. However, data from a cross-sectional study were analysed, and therefore only association and not causality can be determined. Another limitation is the assumption that respondents who are living with HIV will have a greater knowledge of HIVST. A previous study has reported on the strengths and limitations of the study.^18^ The recommendations for increasing the utilisation of HIVST focus on improving education and economic conditions, as these are critical factors that contribute to higher testing rates. Furthermore, integrated strategies that combine socio-economic advancement with tailored media campaigns can effectively increase HIVST uptake among reproductive-aged women in a concise and impactful manner.

## Conclusion

The prevalence of HIVST uptake was low. HIV self-testing was influenced by sociodemographic factors. The findings highlight the importance of addressing women’s needs to know their HIV status as a cornerstone for implementing programmes and research to increase HIVST uptake. The HIVST is recommended for women who would not otherwise test, and it may be critical to achieve the first of the UNAIDS 95-95-95 targets, knowing one’s HIV status.
